# Oral Delivery Mediated RNA Interference of a Carboxylesterase Gene Results in Reduced Resistance to Organophosphorus Insecticides in the Cotton Aphid, *Aphis gossypii* Glover

**DOI:** 10.1371/journal.pone.0102823

**Published:** 2014-08-20

**Authors:** You-Hui Gong, Xin-Rui Yu, Qing-Li Shang, Xue-yan Shi, Xi-Wu Gao

**Affiliations:** 1 Department of Entomology, China Agricultural University, Beijing, China; 2 College of Plant Science and Technology, Jilin University, Changchun, China; Institute of Vegetables and Flowers, Chinese Academy of Agricultural Science, China

## Abstract

**Background:**

RNA interference (RNAi) is an effective tool to examine the function of individual genes. Carboxylesterases (CarE, EC 3.1.1.1) are known to play significant roles in the metabolism of xenobiotic compounds in many insect species. Previous studies in our laboratory found that *CarE* expression was up-regulated in *Aphis gossypii* (Glover) (Hemiptera: Aphididae) adults of both omethoate and malathion resistant strains, indicating the potential involvement of *CarE* in organophosphorus (OP) insecticide resistance. Functional analysis (RNAi) is therefore warranted to investigate the role of *CarE* in *A. gossypii* to OPs resistance.

**Result:**

*CarE* expression in omethoate resistant individuals of *Aphis gossypii* was dramatically suppressed following ingestion of dsRNA-*CarE*. The highest knockdown efficiency (33%) was observed at 72 h after feeding when dsRNA-*CarE* concentration was 100 ng/µL. The CarE activities from the *CarE* knockdown aphids were consistent with the correspondingly significant reduction in *CarE* expression. The CarE activity in the individuals of control aphids was concentrated in the range of 650–900 mOD/per/min, while in the individuals of dsRNA-*CarE*-fed aphids, the CarE activity was concentrated in the range of 500–800 mOD/per/min. In vitro inhibition experiments also demonstrated that total CarE activity in the CarE knockdown aphids decreased significantly as compared to control aphids. Bioassay results of aphids fed dsRNA-*CarE* indicated that suppression of *CarE* expression increased susceptibility to omethoate in individuals of the resistant aphid strains.

**Conclusion:**

The results of this study not only suggest that ingestion of dsRNA through artificial diet could be exploited for functional genomic studies in cotton aphids, but also indicate that *CarE* can be considered as a major target of organophosphorus insecticide (OPs) resistance in *A. gossypii*. Further, our results suggest that the *CarE* would be a propitious target for OPs resistant aphid control, and insect-resistant transgenic plants may be obtained through plant RNAi-mediated silencing of insect *CarE* expression.

## Introduction

Carboxylesterases (CarE, EC 3.1.1.1), or carboxyl/cholinesterases, are known to play significant roles in the metabolism of xenobiotic compounds in many insect species [1]–[2]. Many studies have reported that the elevation of esterase activity through gene amplification or up-regulated transcription accounts for some degree of resistance to insecticides in some insects [3]. This phenomenon is so common that, overexpression of esterases has become a dominant criterion in identifying the development of resistance to organophosphorus insecticides (OPs). This has been well documented in numerous insect species including *Myzus persicae* [4]–[6], *Aphis gossypii* [7]–[9], *Bemisia tabaci* [10], *Culex pipiens quinquefasciatus* [11]–[15], other mosquitoes in *Culicine* [16]–[20], *Nilaparvata lugens* [21], and *Locusta migratoria manilensis* [22]. Metabolic resistance to OPs was also associated with mutations in esterase gene sequences in several insect species. Some mutations (G137D and W251L/S) in esterase genes that confer resistance to OPs have been reported in *Musca domestica* [2], [23]–[25], *Lucilia cuprina* [26]–[27], *Cochliomyia hominivorax* [28] and *Culex pipiens* [29]. Cui et al. [30] reported that G/A151D or W271L mutations could be common mechanisms in the development of OP resistance in Dipteran species.

The cotton aphid, *Aphis gossypii* (Glover) (Hemiptera: Aphididae), is an important pest of a number of agriculturally important crops; not only because of its destructive damage to crops through feeding and virus transmission [31], but also due to its extreme ability to develop resistance to many classes of insecticides, including OPs, pyrethroids, and carbamates [7]–[8]. Elevation of esterase activity through up-regulated esterase transcription, as well as point mutations within esterase genes, which change substrate specificities, are two known mechanisms of esterase-mediated insecticide resistance [9], [32]–[39]. Previous studies in our laboratory found that *CarE* was more highly expressed in the apterous adults of both omethoate- and malathion- resistant *Aphis gossypii* strains as compared to apterous adults from susceptible strains. These findings strongly suggested the potential involvement of *CarE* in OPs resistance in these strains [7]–[9]. As such, more detailed functional analysis investigating the role of *CarE* in OPs resistance in resistant *A. gossypii* strains is warranted.

Post-transcriptional gene silencing by RNA interference (RNAi) is a very useful tool to examine the functions of individual genes. RNAi is mediated by double-stranded RNA (dsRNA) that is cleaved into 21–23 nucleotide small interfering RNAs (siRNAs) by an RNase III-type enzyme known as Dicer [40], [41]. RNAi has been successfully used to investigate gene function in the pea aphid *Acyrthosiphon pisum* and the green peach aphid *Myzus persicae* [42]–[47]. Aphids can be fed artificial diets which are sandwiched between thin parafilm membranes [47]. In *A. pisum*, both microinjection and dsRNA-feeding through artificial diets have been reported to be valuable methods for achieving RNAi [42]–[46]. In *M. persicae*, a plant-mediated RNAi approach was documented to knockdown gene expression by up to 60% in transgenic *Nicotiana benthamiana* and *Arabidopsis thaliana* [47]. RNAi has not yet been employed for gene functional studies in *A. gossypii*. It is difficult to perform microinjections without affecting aphids’ survival rates, as cotton aphids, like *M. persicae*, are smaller than *A. pisum*. As such, dsRNA delivery through feeding may be an efficacious method for RNAi-based functional studies in *A. gossypii*.

In this study, we used artificial diet feeding and RNAi methods to functionally analyze the role of *CarE* in the omethoate resistance of an *A. gossypii* strain known to be resistant to omethoate. We measured aphid susceptibility to omethoate, CarE activity in individual aphids, and the *in vitro* inhibitory effects of S,S,S-tributyl phosphorotrithioate (DEF) on CarE activity in the omethoate resistant aphids 72 h after feeding on ds-*CarE*.

## Materials and Methods

### Insects

The omethoate-resistant aphid strain used in this study is the same strain in which overexpression of *CarE* was previously identified [7]. The resistant strain was initially established from a field population originally collected in 1999 from cotton fields in Xinjiang Uygur Autonomous Region, China. Many generations of this strain were subjected to omethoate selection pressure in our laboratory, using the leaf-dipping method described by Moores et al. [48]. The susceptible strain was supplied by Dr. Donghai Zhang (Shihezi University, Xinjiang Uygur Autonomous Region, China) in 1999, and was maintained over many generations without exposure to insecticides.

### Total RNA isolation, synthesis of cDNA and dsRNA

Apterous adult aphids were homogenized in TRIzol reagent (Invitrogen, USA). The extracted RNA samples were treated with DNase (RNase free) (NEB, USA) to exclude DNA contamination. The total RNA was analyzed with gel electrophoresis and quantified using a spectrophotometer (UV-2550; SHIMADZU, Japan). The first-strand cDNA was synthesized according to the manual of the SuperScript III first-strand synthesis system for RT-PCR kit (Invitrogen, USA). All of the available nucleotide sequences of the *A. gossypii CarE* gene (Genbank No. AY485218 and Genbank No. AY485216) were retrieved from the NCBI GenBank database, and a homology search to define the conserved regions was carried out using Megalign (DNASTAR) software. The forward primer 5′-taatacgactcactataggg TAACCCTTGGGCGTTTACTG-3′and the reverse primer 5′-taatacgactcactataggg GGTCTCGTCGCAAAAATCAT-3′ were used to amplify a *CarE* gene fragment. A 686 bp fragment was amplified and confirmed by sequencing. The fragment was used as a template to generate the corresponding dsRNA using the MEG Ascript RNAi kit (Ambion, USA). dsRNA-*CarE* was dissolved in 50 µL diethypyrocarbonate (DEPC)-treated water, analyzed with gel electrophoresis (1% agarose), and quantified using a spectrophotometer (UV-2550; SHIMADZU, Japan).

### Rearing on artificial diet and dsRNA feeding

**Figure 1:**
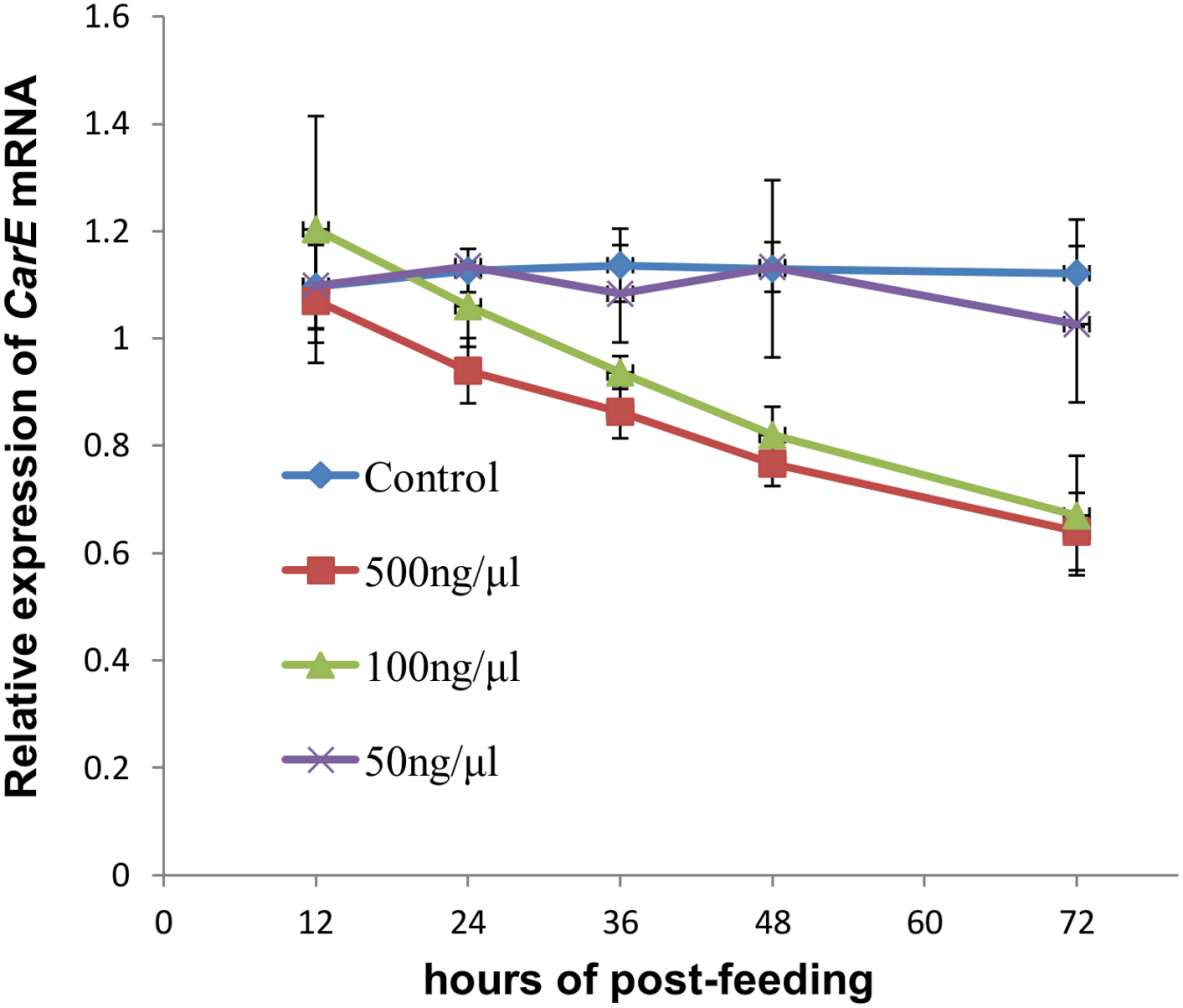
The effect of time course and dsRNA-*CarE* dose on RNAi efficiency of the *Aphis gossypii*. The relative expression of *CarE* mRNA with time course in Aphis gossypii fed on artificial diets with or without *CarE*-dsRNA were recorded. The final concentration of *CarE*-dsRNA in the artificial diet was 50, 100, 500 ng/µL respectively, the artificial diet without *CarE*-dsRNA used as the control. The transcript abundances were determined relative to the normalized calibrator, i.e. cDNA from non-RNAi apterous adults (only fed on cotton leaves), which was set to 1.0. Each treatment had three biological replicates, and 20 insects were used per pooled RNA sample. The results are shown as means ± S.D.

The artificial diet recipe and the rearing device used for this study were developed based on the methods of Mittler [49] with some modifications. The diet was prepared in DEPC-treated water to ensure the absence of RNase activity. For the dsRNA feeding experiments, dsRNA-*CarE* was added into the artificial diet at 50, 100, and 500 ng/µL concentrations. Artificial diet lacking dsRNA-*CarE* was used as a control. Third instar (L3) stage aphids grown on cotton leaves were transferred onto the artificial diet device for rearing. The artificial diet was sealed between two layers of Parafilm in a 2 cm diameter feeding arena; twenty apterous adult aphids were placed in each arena. The arena was covered with a fine mesh to prevent their escape. The insects were reared under controlled growth conditions: 27±1°C, 65±5% relative humidity, and 16∶8 h light:dark photoperiod. After feeding for 24 h, the aphids were collected or transferred onto cotton leaves for the subsequent experiments. In order to determine the optimal dsRNA-*CarE* concentration and the optimal silencing time (different intervals after feeding) to ascertain the maximum silencing efficacy of *CarE*, five silencing times (12 h, 24 h, 36 h, 48 h, and 72 h post-feeding) of each dsRNA-*CarE* concentration (50, 100, and 500 ng/µL) were sampled and evaluated by real time PCR (protocols described below). Based on the results from these optimization studies, aphids fed with dsRNA-*CarE* at the concentration of 100 ng/µL and with the interval of 72 h post-feeding were used for the omethoate toxicity, CarE activity, and *In vitro* CarE inhibition assays. We did not sample at the 500 ng/µL concentration in the formal assays; as the 100 ng/µL dsRNA concentration was found to have the same silencing efficacy as the 500 ng/µL dsRNA concentration at the 72 h post-feeding interval (Figure 1).

### Quantitative real-time PCR (qRT–PCR)

Cotton aphid cDNA was prepared as described in section 2.2, above. The primer pair of CS2 (5′-CATACCCTACGCTCAACCAC-3′) and CA2 (5′-GCAATCTTCACTTCCAACGA-3′) was designed for detecting the transcript levels of *CarE*. The primer pair of R1 (5′-ATTGACGGAAGGGCACC-3′) and R2 (5′-CGCTCCACCAACTAAGAACG-3′) was designed based on the 18S rRNA gene (Genbank No. AF487716), and was used as an internal reference for the relative expression analysis. The qRT-PCR assays were conducted on an ABI 7300 Real time PCR system (ABI) following the manufacturer’s recommendations. The reactions were performed in a 10 µL reaction mixture, which contained 4 µL SYBR, 0.2 µL ROX I, 2.6 µL ddH_2_O, 0.2 µL primers, and 2 µL cDNA (equivalent to 0. 08 µg of total RNA). The cycling parameters were 95°C for 30 s; followed by 40 cycles of 95°C for 5 s; 60°C for 31 s. After the cycling protocol, the final step was applied to all reactions by continuously monitoring fluorescence through the dissociation temperature of the PCR product at a temperature transition rate of 0.1°C/s, to generate a melting curve. Quantification was conducted according to the 2^−ΔCt^ method [50]. The transcript abundances were determined relative to the normalized calibrator, i.e. cDNA from non-RNAi Apterous adults (fed with cotton leaves), which was set to 1.0. The experiment was conducted three times independently, with different RNA preparations. The qRT-PCR results are presented as means with standard errors (SE) of transcript levels, on a logarithmic scale. The statistical significance of changes in gene expression was calculated using a Student’s t-test for all 2-sample comparisons. A value of *P*≤0.05 was considered to be statistically significant (* indicates P≤0.05; ** indicates P≤0.01; *** indicates P≤0.001).

### Susceptibility of aphids to omethoate after RNAi of *CarE*

Omethoate toxicity in the resistant aphids was determined by the leaf-dipping method described by Moores et al. [48] and Cao et al. [7]. Briefly, for the bioassays, a stock of insecticide was prepared in acetone and diluted to a series of six concentrations with distilled water containing 0.05% (v/v) Triton X-100 and 1% acetone. Cotton leaf discs (15-mm diameter) were dipped in omethoate solutions for 5 s, placed in the shade to air dry, and then placed upside down on an agar bed (25 mm in depth) in the wells of 12-well tissue-culture plates. Bioassays were carried out by exposing 45 apterous adults (15 per well) to omethoate-treated leaves. The aphids were confined by applying a ring of fluon to the exposed lip of each well. Assays of each concentration were replicated at least three times, and mortality was assessed at 25°C, 24 h after commencing the treatment. LC_50_ values were calculated with POLO software (LeOra Software Inc., Berkeley CA).

The LC_50_ value of the aphids to omethoate was used as the diagnostic dose for assessment of the sensitivity of cotton aphids to omethoate at 72 h post-feeding of dsRNA-*CarE*. The mortality was assessed at 25°C, 24 h after exposure to omethoate. The control was conducted by using the aphids fed with artificial diet only. This experiment was repeated three times. There were 60 individuals aphids in each replicate. The statistical significance of mortality rate was calculated using a Student’s t-test for all 2-sample comparisons. A value of P≤0.05 was considered to be statistically significant (* indicates P≤0.05; ** indicates P≤0.01; *** indicates P≤0.001).

### CarE activity in individual aphids

For these assays, each adult aphid was homogenized in 100 µL ice-cold phosphate buffer (0.04 M, pH 6.5). The homogenate was centrifuged at 4°C, 10,000 g for 15 min, and the supernatant was used as the enzyme source for measuring the activity of CarE. CarE activity was measured by the method of van Aspern [51] modified for use of a microplate reader (ACT-AMPR-750, ACTGene). 50 µL of homogenate (equivalent to half of one cotton aphid) and 50 µL phosphate buffer (0.04 M, pH 6.5) were added to each well in a microplate; freshly prepared 100 µL mixed solution (6 mg Fast Blue RR in 10 ml 100 µM α-NA) was then added to each well. The absorbance (405 nm) was read 30 times over the course of 5 min. The slope, OD increase value per min per aphid, was taken to represent the CarE activity of a single aphid. 100 cotton aphids were analyzed for both the dsRNA-*CarE* fed treatment group and the control group.

### *In vitro* CarE inhibition by DEF

S,S,S-tributyl phosphorotrithioate (DEF) is an inhibitor of CarE. It can inhibit the activity of CarE *in vitro*. The IC_50_ of DEF was determined according to the method of Young et al [52] with modifications. For these assays, one hundred apterous adults with similar color and size from 72 h post-feeding of dsRNA-*CarE* group and the control group were homogenized in 1 mL of ice-cold phosphate buffer (0.04 M, pH 7.0). The controls were aphids fed with artificial diet only. The homogenates were centrifuged at 4°C, 10,000 g for 15 min, The supernatant was used as an enzyme source for measuring the activity of CarE. Stock solutions (30 mM) of DEF were prepared in acetone, and serial dilutions of DEF solutions from 0.156 to 10 mM were prepared in ice-cold phosphate buffer (0.04 M, pH 7.0). Enzyme solutions in buffer, and buffer only served as positive and negative treatments, respectively. 50 µL of insect homogenate was incubated for 30 min with 5 µL DEF solution. 450 µL of phosphate buffer (pH 7.0, 0.04 M) and 1.8 mL 0.3 mM substrate solution (α-NA) were then added. The reaction was stopped by the addition of 0.9 mL of stop solution (two parts of 1% Fast Blue BB and five parts of 5% sodium dodecyl sulfate) after incubation at 30°C for 15 min. The color was allowed to develop for 15 min at room temperature, and the absorbance was measured at 600 nm for α-NA with a UV/VIS Spectrometer Lambda Bio40 (Perkin-Elmer, USA). Mean levels of residual esterase activity after inhibition with DEF were based on protein content and α-NA standard curves. Protein content was determined by the method of Bradford [53], using bovine serum albumin as the standard. The IC_50_ was calculated based on the concentration of DEF and the value of the residual CarE activity.

## Results

### *CarE* -dsRNA synthesis and dose effect and time course on RNA interference

**Figure 2:**
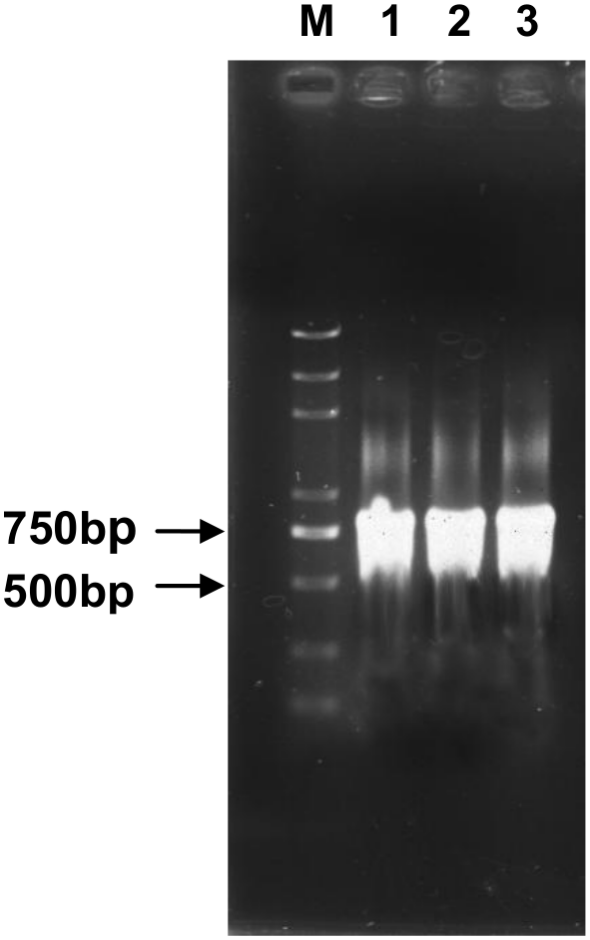
Electrophorosis of dsRNA-*CarE*. M: Molecular weight marker 5000; 1, 2, 3: dsRNA-*CarE*. Three groups of dsRNA-*CarE* were dissolved in 50 µL diethypyrocarbonate (DEPC)-treated water, analyzed with gel electrophoresis (1% agarose).

In this study, A 686 bp *CarE* gene fragment was amplified and confirmed by sequencing. Figure 2 shows a 686 bp single band of dsRNA-*CarE* was amplified using a 686 bp *CarE* gene fragment as a template. The final concentration of the three groups of dsRNA-*CarE* (Figure 2) was 780 ng/µl,831 ng/µl and 867 ng/µl, respectively, after dissolution in 50 µL of diethypyrocarbonate (DEPC)-treated water.

**Figure 3:**
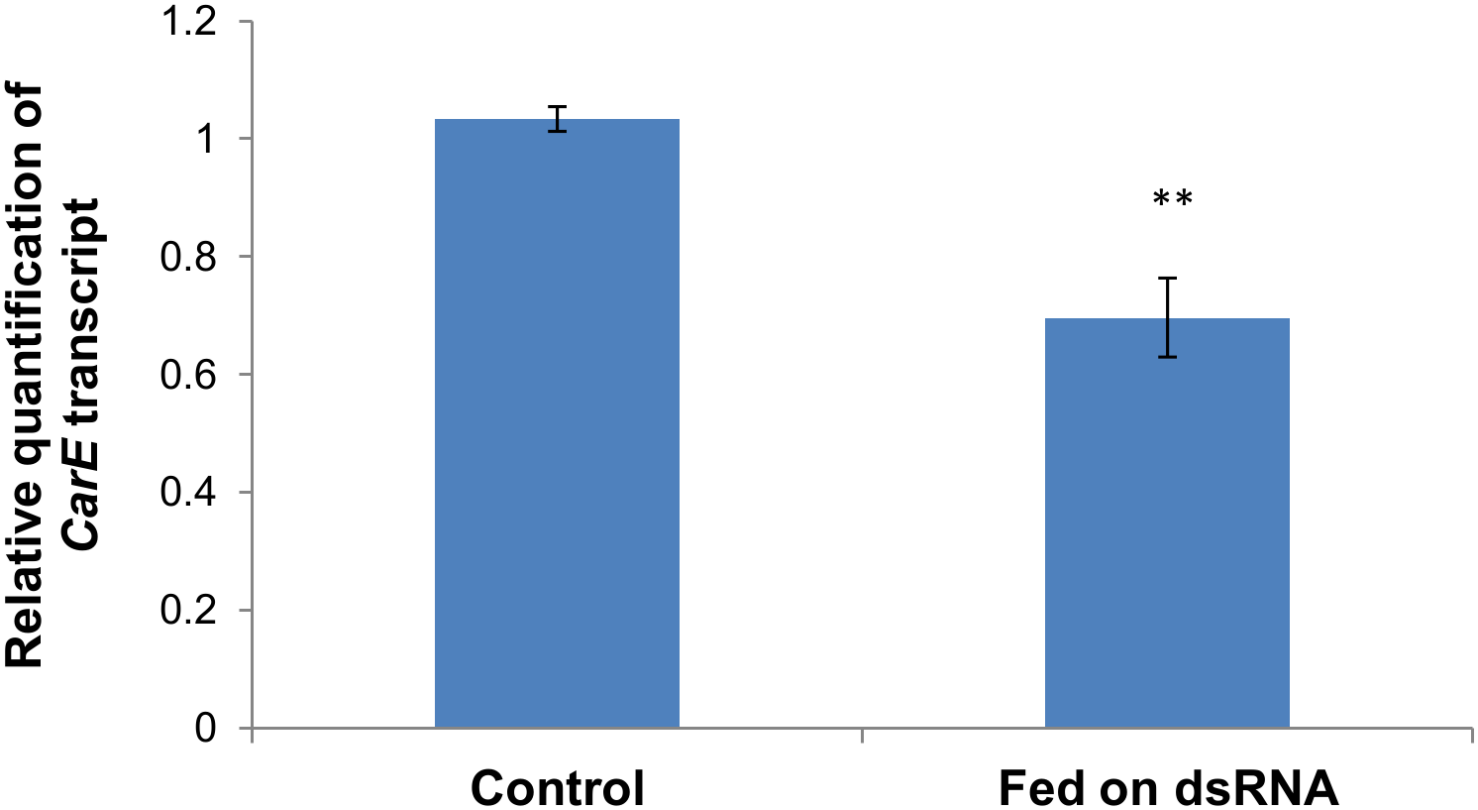
The dsRNA-mediated suppression of *CarE* transcript expression in aphids fed on the artificial diet with dsRNA. The final concentration of dsRNA in the artificial diet was 100 ng/µL. The transcript abundances were determined relative to the normalized calibrator, i.e. cDNA from non-RNAi apterous adults (fed on cotton leaves), which was set to 1.0. The values are means with standard errors of three biological replicates from RNA samples of 20 aphids. “**” indicates significant differences as determined by Student’s t-test (P<0.01).

As mentioned in section 2.3, in order to determine the optimal dsRNA-*CarE* concentration and the optimal silencing time (different intervals after feeding) as well as to ascertain the maximum silencing efficacy of *CarE*, five silencing times (12 h, 24 h, 36 h, 48 h, and 72 h post-feeding) of each dsRNA-*CarE* concentration (50, 100, and 500 ng/µL) were sampled and evaluated by real time PCR. Figure 1 shows that the *CarE*-mRNA expression decreased as ds-RNA-*CarE* concentration increased. The effect at the 500 ng/µl level showed no significant difference from the 100 ng/µl level. At each increase of post feeding times, the *CarE* transcripts expression decreased, with the lowest expression level at post feeding 72 h. Finally, based on the results from these optimization studies, aphids fed with dsRNA-*CarE* at a concentration of 100 ng/µL 72 h post-feeding were examined again for *CarE* transcripts expression. Figure 3 shows that the expression level of *CarE* was significantly lower at 72 h post-feeding in the omethoate- resistant aphids which were fed 100 ng/µL dsRNA-*CarE*, as compared to the control aphids (Student’s t test, t = 4.8, 4 degrees of freedom, P = 0.009). The average reduction of *CarE* expression observed was about 33% (Figure 3), indicating that the dsRNA-mediated knock down of *CarE* transcripts was successful and these silencing conditions can be used for following experiments.

### *CarE* knockdown increases sensitivity to omethoate in the aphids of the resistant strain

**Figure 4:**
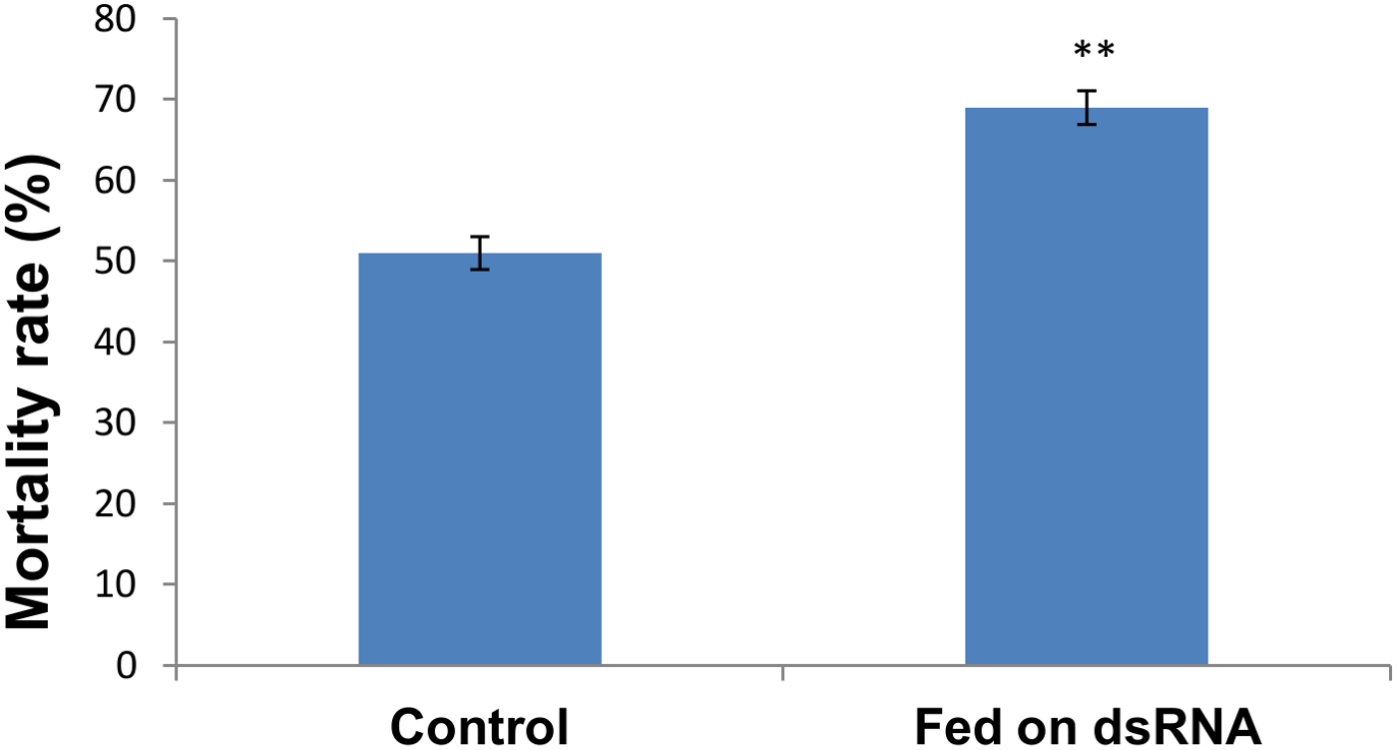
*CarE* knockdown increases resistant aphids’ sensitivity to omethoate. The final concentration of dsRNA in the artificial diet was 100 ng/µL; the artificial diet lacking dsRNA was used as control. The mortality was recorded after 24 h exposure to omethoate (3 replicates, 60 individuals for each replicate). The values are presented as the means with standard errors. “**” indicates significant difference as determined by Student’s t-test (P<0.01).

A Probit analysis of the susceptibility of omethoate resistant aphids exposed to omethoate is summarized in Table 1. The LC_50_ value of the *A. gossypii* resistant strain was 5874 mg/L, and this value was used as the dosage to evaluate the effect of RNAi of *CarE* on the susceptibility of cotton aphids to omethoate. The results demonstrated that mortality increased significantly (Student’s t test, t = −6.182, 4 degrees of freedom, P = 0.003), from 50.78% in the control aphids to 68.44% in the dsRNA-CarE-fed aphids (Figure 4).

**Table 1:** Susceptibility of cotton aphids to omethoate.

Strain	LC_50_ (mg/L) (95% CL^a^)	Slope±SE^b^	?^2^
Resistant strain	5874 (4027.83–8912.50)	2.51±0.41	2.67

a CL: Confidence limited.

b SE: Standard error.

### *CarE* knockdown decreases CarE activity in individual aphids

**Figure 5:**
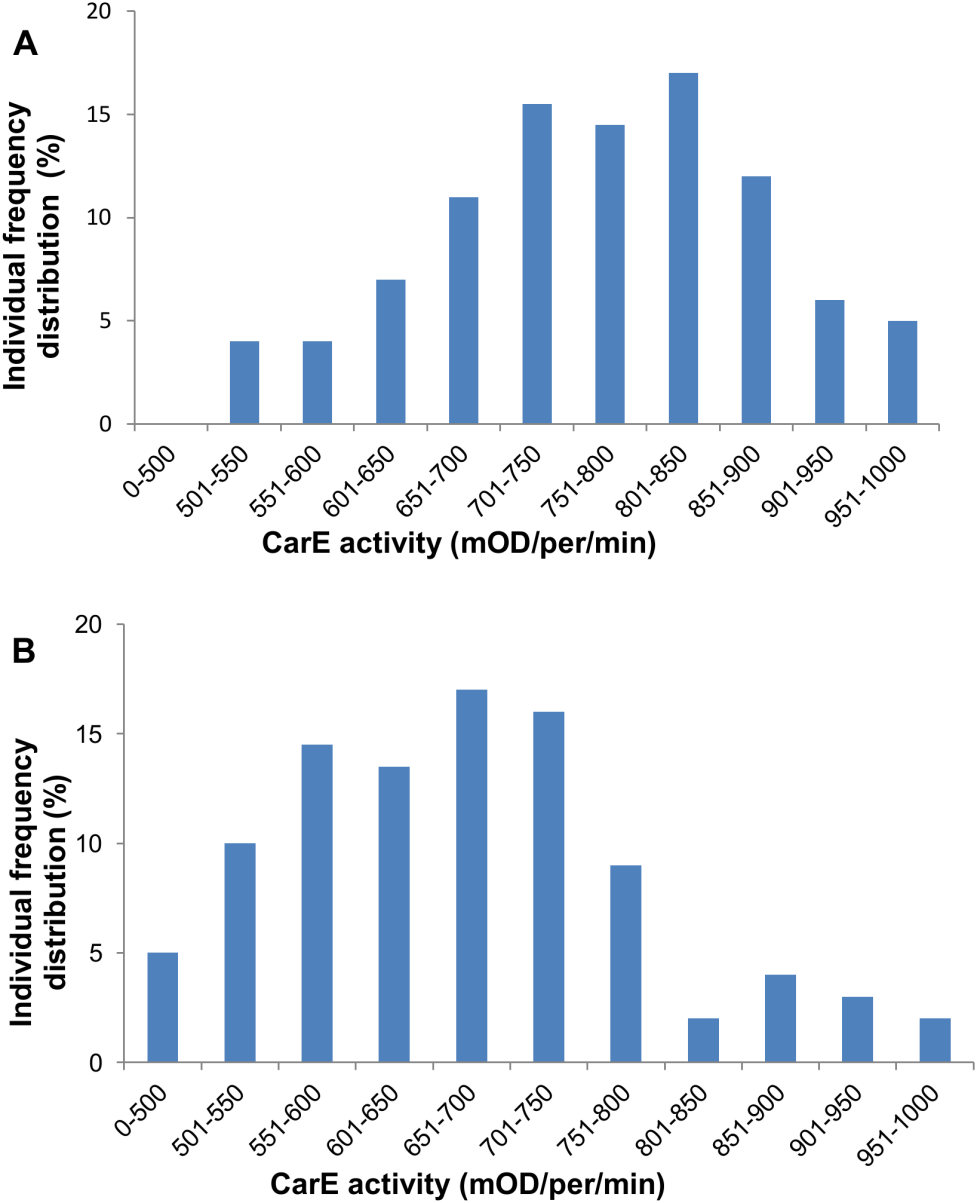
Frequency distribution of individual CarE activity in aphids fed on artificial diet lacking dsRNA-*CarE* or with dsRNA-*CarE*. Total 100 survival aphids were tested at 72 h after feeding on the artificial diet lacking dsRNA of *CarE* (A) or with dsRNA of *CarE* (B). The final concentration of dsRNA in the artificial diet was 100 ng/µL.

The CarE activities of individual aphids were grouped into different activity intervals; each interval level increased by 50 mOD/per/min (Figure 5). Frequency distributions of individuals on each interval level were then calculated for both the control and the dsRNA-fed aphids based on their CarE activities. The CarE activity in the control aphids was concentrated in the range of 650–900 mOD/per/min, with an average CarE activity of 742 mOD/per/min (Figure 5A), while the CarE activity in the dsRNA-fed aphids was concentrated in the range of 500–800 mOD/per/min with an average CarE activity of 677 mOD/per/min (Figure 5B).

### *CarE* knockdown decreases the IC_50_ value of DEF inhibited CarE activity in resistant aphids

The IC_50_ value of DEF for inhibiting CarE activity was 1.2-fold higher in the control aphids than in the dsRNA-CarE-fed aphids (Table 2) and there was a significant difference (Student’s t test, t = 3.771, 4 degrees of freedom, P = 0.020). This result illustrated that CarE activity decreased due to the suppression of *CarE* transcript expression in the dsRNA-*CarE* treated aphids.

**Table 2:** CarE knockdown decreases the IC_50_ value of DEF for inhibiting CarE activity in resistant aphids.

Inhibitor	IC_50_ (mol/L)	
	fed on artificial diet	fed on dsRNA
DEF	0.094±0.003	0.078±0.003*

The values are presented as the means with standard errors. The asterisk indicates significant differences as determined by Student’s t-test (*P*≤0.05). The final concentration of dsRNA in the artificial diet was 100 ng/µL.

## Discussion

Both microinjection and oral delivery of dsRNA through artificial diets have been reported to be valuable methods for achieving RNAi in aphids [42]–[44], [46]. However, there is evidence that micro-injection of dsRNA can cause negative impacts on the survival of the aphids [43]. These impacts may result from mechanical damage or from the sudden higher levels of the injected dsRNA in the hemolymph [47]. Cotton aphids are smaller than *A. pisum,* and it is difficult to do microinjections on cotton aphids without deleteriously affecting aphid survival rates. Therefore, in our study, we used an artificial diet containing dsRNA-*CarE* to knock down the expression of the cotton aphid *CarE* gene. The survival rate of the cotton aphids fed on dsRNA for 24 h was above 90% in our experiment (data not shown), so the delivery of dsRNA via artificial diet can now be considered to be a suitable method for achieving RNAi in this pest. qRT-PCR analyses showed that *CarE* transcript levels in aphids significantly decreased by up to 33% at 72 h after feeding the dsRNA-*CarE* at a 100 ng/µL concentration, as compared to the control group (Figure 3). This is the first example of RNAi in a cotton aphid. The *CarE* transcript level dropped gradually, over time after feeding (Figure 1), as was observed in the light brown apple moth by Turner et al. [54]. This may indicate that quite time is required for the dsRNA to enter midgut cells and/or for the induction of the RNAi process [54]. Although the silencing observed through RNAi in the present study is significant, it was not complete. This result is similar to the RNAi effect observed for the pea aphid [46]. This could be due to the degradation of dsRNA in the artificial diet prior to ingestion, or in the body of aphids. Recently, Allen and Walker [55] reported that the dsRNA present in the saliva of *Lygus lineolaris* is capable of degrading dsRNA. Yu et al. [56] also found that aphids feeding on an artificial diet containing dsGFP caused degradation of that dsRNA in the diet. In summary, our results demonstrate that RNAi by dsRNA feeding is possible in the *A. gossypii,* as it is in nematodes [57], ticks [58], *Epiphyas postvittana* [54] and *A. pisum* [45]–[46]. Oral delivery of dsRNA offers many advantages compared with injection and soaking. It is labor-saving, cost-effective, easy to perform, and applicable for high-throughput gene screening [59]. It is also a less invasive method than microinjection forinducing RNAi in small insects such as aphids and in first and second-instar larvae or nymphs [60].

In insects, CarEs are key components of defense against xenobiotic compounds, including insecticides [1]. CarE-based metabolic resistance to OPs has been observed in many insects, including cotton aphids [7]–[9]. The molecular mechanism of this resistance originates either from mutations in esterase-encoding sequences or from increased transcription of esterase genes [3], [30]. RNAi is a useful tool to probe the functions of genes. To date, there are no reports analyzing the functions of *CarE* genes in *A. gossypii* using RNAi methods. In our study, the LC_50_ dosage of the resistant cotton aphid strain was used to detect the susceptibility of dsRNA-*CarE*-fed cotton aphids to omethoate (Table 1). The mortality of dsRNA-*CarE*-fed aphids was 68.44%, while that of control aphids was 50.78% (Figure 4), indicating that RNAi of *CarE* expression reduced the detoxification metabolic effect of CarE to omethoate. This effect may be caused by reduced total CarE activity. This is consistent with a previous report that overexpression of a *CarE* gene was involved in the resistance to OPs in a omethoate-resistant strain of *A. gossypii* [7]. Our results from the individual aphid CarE activity assays confirm to this as well. We found that the CarE activity in the control aphids was concentrated in the range of 650–900 mOD/per/min with an average CarE activity of 742 mOD/per/min (Figure 5A), while the CarE activity in the dsRNA-*CarE*-fed aphids was concentrated in the range of 500–800 mOD/per/min with an average CarE activity of 677 mOD/per/min (Figure 5B). The results showed significant reduction of CarE activity by dsRNA-*CarE* feeding. This reduction in activity likely reduces the insecticide detoxification ability of the aphids. This conclusion was further supported by the results of *in vitro* CarE inhibition assays, in which the IC_50_ value of DEF was 1.2-fold higher in control aphids than in dsRNA-fed aphids (Table 2). In our study, the susceptibility of aphids to omethoate and enzyme assays after *CarE* knock down implied that the CarE plays an important role in OP resistance.

Feeding of dsRNA targeting vATPase transcripts from an artificial diet achieved a 30% decrease in transcripts of *A. pisum* and caused a significant increase in aphid mortality [47]. Since our results indicated that the suppression of *CarE* transcript levels increased the resistant cotton aphids’ susceptibility to omethoate (Figure 4), it will be appealing to use dsRNA targeting of the *CarE* gene for controlling the OP resistant aphids, and possibly even other pest insects that have similar resistance mechanisms. The plant-mediated RNAi method has been used to effectively silence genes of Lepidopteran, Coleopteran, and Hemipteran insect species [61]–[64]. Researchers have noted that phloem sap-sucking insects, such as aphids, whiteflies, planthoppers and plant bugs, have evolved from minor pests to major pests. It would be a revolution in plant protection if plant-mediated RNAi can be used extensively to protect plants from sucking insect pests [65]. Fortunately, plant-mediated RNAi approaches were documented to knock down *M. persicae* gene expression by up to 60% on transgenic *Nicotiana benthamiana* and *A*rabidopsis *thaliana* [48]. Therefore, plant-mediated RNAi method targeting of *CarE* may be possible and useful for the control of OP resistant aphid pests, and this warrants further investigation in the future.

## Conclusion

In conclusion, our findings provide insights about the role of *CarE* in the xenobiotic metabolism of an OP resistant *A. gossypii* strain. These results suggest that feeding of dsRNA through artificial diet can be exploited for functional studies in cotton aphids. Further, our results suggest that the *CarE* would be a promising potential target for OPs resistance management and aphid control.
